# An Individual Finger Gesture Recognition System Based on Motion-Intent Analysis Using Mechanomyogram Signal

**DOI:** 10.3389/fneur.2017.00573

**Published:** 2017-11-08

**Authors:** Huijun Ding, Qing He, Yongjin Zhou, Guo Dan, Song Cui

**Affiliations:** ^1^Guangdong Key Laboratory for Biomedical Measurements and Ultrasound Imaging, School of Biomedical Engineering, Health Science Center, Shenzhen University, Guangdong, China; ^2^Center for Neurorehabilitation, Shenzhen Institute of Neuroscience, Guangdong, China; ^3^Institute of High Performance Computing, Singapore, Singapore

**Keywords:** mechanomyogram, inertial sensor, finger gesture recognition, motion intent, feature selection

## Abstract

Motion-intent-based finger gesture recognition systems are crucial for many applications such as prosthesis control, sign language recognition, wearable rehabilitation system, and human–computer interaction. In this article, a motion-intent-based finger gesture recognition system is designed to correctly identify the tapping of every finger for the first time. Two auto-event annotation algorithms are firstly applied and evaluated for detecting the finger tapping frame. Based on the truncated signals, the Wavelet packet transform (WPT) coefficients are calculated and compressed as the features, followed by a feature selection method that is able to improve the performance by optimizing the feature set. Finally, three popular classifiers including naive Bayes (NBC), K-nearest neighbor (KNN), and support vector machine (SVM) are applied and evaluated. The recognition accuracy can be achieved up to 94%. The design and the architecture of the system are presented with full system characterization results.

## 1 Introduction

Modeling and recognizing human hand gesture is an extremely important research topic, and it is the core of any intelligent human–computer interaction system with applications in automatic control, virtual reality, augmented reality, human–robotic interaction, and computer animation. In addition, it attracts more attentions and interests in biomedical engineering recently, e.g., prosthesis control and wearable rehabilitation system. As it is difficult to model the gesture accurately from images and the gesture appearance varies a lot, it is still a difficult task to recognize and track hand gestures. On the other hand, stroke is the leading cause of disability in adults worldwide. Exercise disorders as the most common sequelae of stroke can seriously affect normal activity and quality of life. Exercise and training have long been used to restore motor function after stroke. Among that, hand movement as a kind of fine action is more difficult to recover. Finger gesture recognition technique can be used to train and lead the patients to do the rehabilitation exercises, which normally requires real-time interaction and long-term monitoring. Thus, portable system with real-time processing technique is a valuable topic to pay attention and be explored in the stroke application area.

Currently, most hand-gesture recognition systems are using either hand-motion-based (HMB) technologies or motion-intent-based (MIB) technologies. HMB methods are capable of directly detecting the tracking of hand motions. Non-skin attached sensors such as optical sensors, inertial sensors, and force sensors (1) are usually deployed in HMB technologies. Many commercial products, such as Kinect and Data Glove, are built on HMB technologies. However, the performance of HMB-based hand-gesture recognition system degrades when the lighting variations present. The recognition accuracy will also drop significantly if skin colors are too similar to background colors or the usage is in dark environment. In addition, the coverage of the system is limited by the sensors’ measurement range and attaching sensors to fingers and hands make user hand movements unsmoothly and uncomfortable.

MIB methods measure the motion intentions instead of the actual hand motions, which is critical in certain applications such as prosthesis control, virtual reality, and motor rehabilitation. Hand-gesture recognition systems relying on MIB technologies are capable of recognizing the hand gestures based on the forearm muscle activities (2) or the signal decoding from brain (3). The brain related methods are driven by the neuroplasticity for stroke rehabilitation and are explained more from the nervous system. Brain cortex activities are analyzed by imaging techniques, e.g., electroencephalography (4, 5), electrocorticography (6), near-infrared spectroscopy (7, 8), magnetic resonance imaging (9), and optical tomography (10). A review of hybrid brain–computer interface techniques can also be found in Ref. (11). All these methods by using imaging technologies are with high cost and sensitive to the experimental environment and setup. For example, electroencephalography is not reliable under exposure to high-intensity magnetic fields and also cannot be applied to the participants having metal implants in their body (12).

The most commonly used forearm muscle analysis solutions relying on MIB approach are the surface electromyography (sEMG) method and the mechanomyogram (MMG) method. This kind of methods explains more about the efforts made by muscles. sEMG uses the surface electrodes to record the electrical currents signal produced by the muscular contraction and many sEMG-based systems have been proposed in the past decades (13). On the other hand, the MMG approach becomes an active topic recently because these sensors can be made into small sizes with reduced cost and good performances. MMG is a low-frequency mechanical signal which can be detected during the muscular contracting period. It is transmitted from the muscle to the surface skin by the soft tissue, and it can be detected by the motion sensors, i.e., inertial sensors (14, 15), laser sensors (16, 17), and microphones (18, 19) attached to the skin. Among all the sensors, inertial sensors are widely used. They are cheaper and more wearable than laser sensors (20). For the microphone-based MMG acquisition system, an air chamber is normally required to be placed between the condenser microphone and the surface of the skin to improve the quality of the acoustic signal. The design of the air chamber is a key factor which affects the frequency response of the acquisition system (19). In addition, the microphone-based MMG acquisition system is less wearable than inertial sensor-based system. Since different hand gestures are due to different modes of voluntary isometric contraction (17), it is possible to recognize the hand gestures based on the MMG signal detected from the forearm muscle group (14, 21). Compared with the sEMG approaches, MMG approaches have some advantages. First of all, MMG provides flexibility in setting up the sensors. Since the MMG signal can be detected on the distal of soft tissue during the muscle contraction (14), the location for placing the sensors are more flexible in contrast to the sEMG, which normally requires an experienced technician to find out the good positions for the sEMG sensors. This flexibility will give MMG users better user experiences compared with sEMG users. Second, the MMG signal is independent of the skin impedance. On the other hand, the sEMG signal is easier to be affected by the skin impedance, which is one of the shortcomings of sEMG-based approach (22). This drawback makes the sEMG sensors unstable due to the reasons that the skin impedance is sensitive to many factors such as temperature and humidity. Finally, systems with MMG have low cost and have less computational loads. MMG is a low-frequency signal with the range of 10–22 Hz (23) while sEMG signal is in the range of 50–900 Hz (15). In that case, there is no need for high-frequency electronic components in the MMG acquisition system and lower sampling rate produces less data for processing thus reduces the computational load.

Due to these advantages, many hand-gesture identification systems are built using MMG–MIB approach. A recognition system is built for identifying the flexion and extensor of the wrist in 1986 (14). The MMG signal was collected by a microphone from the flexion digitorum and extensor digitorum in the system. The amplitude of the MMG signal is used to classify these two gestures. Based on a similar idea, a recognition system is built and capable of identifying additional hand gestures including wrist flex, wrist extensor, hand open, and hand close (24). In this system, the MMG capture system is deploying acceleration sensors. Then, wavelet packet transform (WPT) is used to process the MMG raw signal, followed by the singular value decomposition (SVD) to reduce dimensions of features for gesture recognition. Based on a linear discriminant analysis classifier, the identification algorithm gives an average accuracy rate of 89.7%. After that, many researchers (25–27) extend the study for the recognition of different hand gestures.

There are only few reported MMG–MIB systems on finger gesture recognition (FGR) in the literature. Three types of finger gestures including thumb flexion, pinkie flexion, and middle three finger flexions are classified in an FGR system based on the microphone-accelerometer (28). This system recognizes these finger gestures by root mean square (RMS) of the MMG signal amplitudes, and its average identification accuracy rate is 76.2%. However, individual flexion of the middle three fingers cannot be distinguished in this FGR system. Subsequently, a system is presented in Ref. (29) to identify the middle three finger gestures, namely, index tapping, middle tapping, and ring finger tapping. Two tri-axial accelerometers are used to record the MMG signal from the forearm muscles. Three types of finger gestures can be recognized, and the average accuracy rate is 75%. At the same time, some researchers dedicate to recognize more motion patterns of a single finger. For instance, a system proposed in Ref. (30) is capable of classifying four thumb motion patterns, which includes flexion, extension, abduction, and adduction. The features are extracted by a hybrid algorithm combing the mean absolute value of MMG signal, RMS of amplitude, mean frequency, etc. The average accuracy rate is 81.5% achieved by the quadratic discriminant analysis.

Among all the existing FGR systems, none of them can recognize the individual movement of five fingers, which is the goal of our efforts. Our previous work (31) proves that the individual finger tapping can be recognized based on the analysis of forearm muscle contractions, and a support vector machine (SVM) classifier shows a good performance on classification and recognition. In this article, we further present a FGR system capable of recognizing thumb tapping, index finger tapping, middle finger tapping, ring finger tapping and little finger tapping based on MMG-MIB approach. Two auto-event annotation algorithms are applied and evaluated for detecting the finger tapping frame. They are better for real-time processing system than the manual cutting approach although the latter one is more accurate. Based on the analysis of MMG frame, the wavelet packet transform (WPT) coefficients are calculated and compressed as the feature, followed by a feature selection method which is able to improve the performance of only one kind of feature. Finally, three popular classifiers including NBC, KNN, and SVM are applied and evaluated by their performances on finger gesture recognition.

In the current stage, only healthy participants are invited to attend our experiments to prove the feasibility of recognition for individual finger movement. For stroke patients, the situation is extremely complicated. The applicable subjects for the experiments of the proposed system are limited to the group of who is capable of controlling the contractions of the forearm muscle. Finding such subjects and to some extent further studying how the proposed system can be adaptive and scalable for subjects with different degrees of the capability of controlling forearm muscle is a much broader topic demanding more effort which will be further explored in future work.

## 2 System Architecture

**Figure 1:**
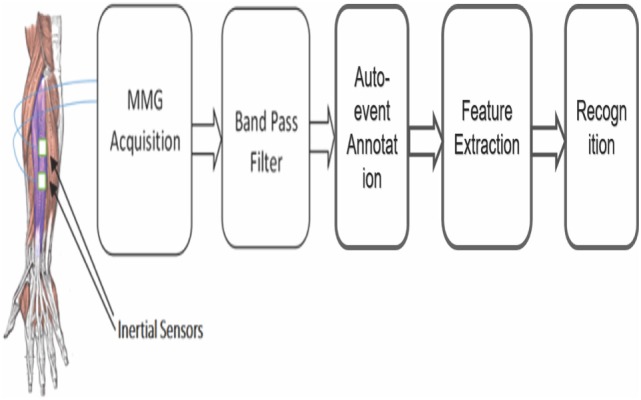
The flowchart of the proposed FGR system.

The proposed system relies on two MMG signal channels for identifying movements of five fingers. The architect of the proposed FGR system is shown in Figure 1. The first module contains the MMG acquisition system obtaining the two-channel MMG signals from the forearm muscle by the inertial sensor. Then, the detected MMG signals are going through a band-pass filter to reduce noise distortion. The third stage of the system consists of the tapping event detection (TED) algorithm to extract the MMG signal segments with the muscle activity information. The next stage is the feature extraction process on the obtained MMG signal segments. Finally, different classifiers including SVM, KNN, and NBC are built for the recognition purpose based on the features extracted. A detailed description of each sub-system is given below.

### 2.1 MMG Acquisition Module

The MMG signal is detected by the inertial sensor MUP6050 (InvenSense, USA), which is capable of capturing the information of acceleration and angular velocity. In our system, the acceleration signal from its Z-axis is recorded for FGR. Since Z-axis is perpendicular to the skin surface, it is the most important direction for studying the motions of muscular contraction (24). As mentioned previously, the MMG signals measuring the mechanical activities are characterized by low-frequency vibrations (<50 Hz) (32). The sampling frequency is chosen to be 1 kHz, and the cutoff frequency is chosen to be 200 Hz for the embedded analog to digital converter and low-pass filter in MUP6050, respectively. Since the contractions of the extensor digitorum muscle are the main muscle activities when finger taps, the MMG signal is detected from the belly of extensor digitorum muscle. Two sensors are attached on the skin surface within the above mentioned area to have a multichannel understanding of the extensor digitorum muscle. This is the reason why we have two-channel MMG signal measurements.

The relatively high movement artifact is the major deficiency of MMG acquisition, which can significantly degrade the recognition accuracy. Thus, all the subjects were asked to put their arms on the desk to keep their forearms as motionless as possible during the experiments.

### 2.2 Band-Pass Filtering Module

As reported in Ref. (17, 33), the predominant power in MMG signals detected is within 10–22 Hz, and the acoustic frequency contents of MMG signal produced by contracting muscles are within 5–50 Hz (32). Thus, a 4th-order band-pass Butterworth filter in the range of 0.1–50 Hz is used in preprocessing to retain the useful information and reduce noises.

### 2.3 Auto-Event Annotation Module

The MMG signals after band-pass filters need to go through our auto-event annotation process. Auto-event annotation is designed to detect the finger tapping event and extract the activity segment in this article. Thus it is also called tapping event detection (TED) in the following. This module is also considered as the first stage of finger gesture recognition. In the literature, the MMG signal segments are obtained by manual cut (14) or predefined time slot when the participants are asked to make each movement (24, 29). These approaches introduce the inconvenience in either the data preprocessing stage or in the data acquisition process. Furthermore, they do not have the capability of real-time processing. To make the proposed FGR system with real-time processing capability, an automatic TED algorithm is required. In our system, two different TED algorithms are tested. The first one is the root mean square (RMS) algorithm (30), and the other algorithm is the difference-template filtering (DTF). Their performances are compared in our experiments.

#### 2.3.1 Root Mean Square (RMS) Algorithm

The RMS algorithm has been used in many MMG signal processing applications such as the assessment of muscle function (32, 34) and automatic detection of muscle activities (35). It can be described by the following equation:

(1) $$Z_{r}\left(t\right)=\left\{\begin{array}{cc} 1,\; & \mathrm{if}\;\left(g_{r}\left[t\right]>TH_{r}\right)\\ 0,\; & \mathrm{otherwise} \end{array}\right.,$$

where *t* is the time index. *Z_r_*[*t*] is the output of this algorithm. Its value will be set to 1 when the finger motion event is detected, otherwise it will be zero. *g_r_*[*t*] and *TH_r_* are defined by the following equations:

(2) $$g_{r}[t]=\sqrt{\frac{1}{W}\sum_{i=t}^{t+W}x{\left[i\right]}^{2},}$$

(3) $$TH_{r}=\alpha_{r}*\frac{\sum_{i=T_{s1}}^{T_{s2}}\;g_{r}\left[i\right]}{T_{s2}-T_{s1}},$$

where the *g_r_*[*t*] is the RMS value from *x*[*t*] to *x*[*t* + *W*] and the parameter *W* is the window size. As reported in Ref. (35), an appropriate analysis window size for MMG signal is from 100 to 400 ms. In our system, the window size is fixed to 400 ms, which has a superior performance in contrast to other smaller sizes. The parameters, *T_s_*_1_ and *T_s_*_2_, are the start and end points of time during which the forearm muscle group of participants are required to keep relaxed. The parameter, *α_r_*, is the threshold scaling factor which is set to 2.15 in our system.

#### 2.3.2 Difference-Template Filtering (DTF)

A DTF-based TED algorithm is proposed to detect the muscle activities in our system. The vector *D* = [−1, −1, −1, −1, −1, −1, −1, −1, 0, 1, 1, 1, 1, 1, 1, 1, 1] is designed as the difference template for convolution with MMG signal. The difference template-based TED can be described by equations (4)–(6):

(4) $$Z_{d}\left(t\right)=\left\{\begin{array}{cc} 1,\; & \mathrm{if}\;\left(g_{d}\left[t\right]>TH_{d}\right)\\ 0,\; & \mathrm{otherwise} \end{array}\right.,$$

where *g_d_*[*t*] is defined by equation (5) and the parameter *TH_d_* is defined by equation (6).

(5) $$g_{d}=D⊗x.$$

The ⊗ is the convolution operator, and *x* is the input MMG signal.

(6) $$TH_{d}=\alpha_{d}*\frac{\sum_{i=T_{s1}}^{T_{s2}}\;g_{d}\left[i\right]}{T_{s2}-T_{s1}}.$$

The parameter *α_d_* in above equation is the threshold scaling factor, which is set to 2 in our system. *T_s_*_1_ and *T_s_*_2_ have same definitions as mentioned above.

### 2.4 Feature Extraction Module

Feature extraction is an extremely important process in our system as the recognition accuracy is heavily dependent on whether indicative and relevant features to finger movements can be found. In our system, the MMG signals are transformed to wavelet domain for wavelet packet transform (WPT) coefficients. Then singular value decomposition (SVD) is applied to reduce the dimension of the coefficients for computational efficiency. Then, WPT features will be sent to our classification module for the recognition purpose.

#### 2.4.1 Feature Matrix Extraction

Wavelet transform (WT) is proposed for multi-resolution analysis developed from the Fourier transform. It is able to represent the local signal characteristics in time-frequency domain. However, its resolution is decreasing when the signal frequency is increasing. In other words, the resolution in high-frequency region is very poor in the WT analysis. As an extension of the standard WT, WPT is able to provide an arbitrary time-frequency resolution (36). Therefore, WPT is used to extract the signal characteristics from the MMG signal here for analyzing the muscle activities.

**Figure 2:**
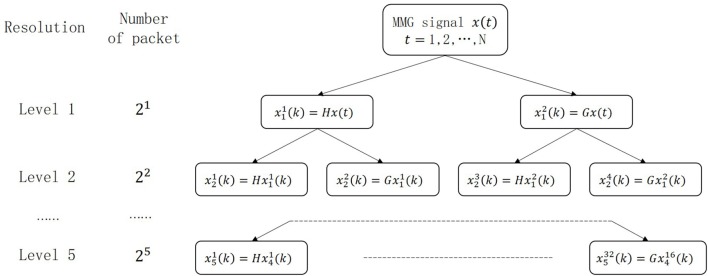
The wavelet packet decomposition tree.

The Figure 2 shows a 5-level wavelet packet decomposition which is also known as optimal subband tree structuring. Each node of the tree is marked as $x_{j}^{p}\left(t\right)$, where *j* is the level with the range from 1 to 5, and *p* = 1, … , 2*^j^* is the number of the packet in the *j*th level. Each node, $x_{j}^{p}\left(t\right)$, can be decomposed to two nodes, $x_{j+1}^{2p-1}\left(t\right)$ and $x_{j+1}^{2p}\left(t\right)$, which is described in equation (7). The reconstructions are given in equations (8) and (9) as below:

(7) $$X_{j}^{p}=X_{j+1}^{2p-1}⊕X_{j+1}^{2p},$$

(8) $$x_{j}^{2p-1}\left(t\right)=\sqrt{2}\sum h\left(n\right)x_{j-1}^{p}\left(n-2t\right),$$

(9) $$x_{j}^{2p}\left(t\right)=\sqrt{2}\sum g\left(n\right)x_{j-1}^{p}\left(n-2t\right),$$

where *n* and *t* are both time indexes, *h*(*n*) in equation (8) is the scaling function, *g*(*n*) in equation (9) is the wavelet filter, and the details of *h*(*n*) and *g*(*n*) can be referred to Ref. (37). In our system, every MMG signal segment is decomposed into the 5th level shown in Figure 2. Each node of the 5th level wavelet is a column vector containing *LN*/32 coefficients, where *LN* is the size of the processed MMG signal. All the nodes of the 5th level construct a (*LN*/32) x 32 feature matrix, *X*, shown in the following equation:

(10) $$X=\left[x_{5}^{1}\left(k\right),x_{5}^{2}\left(k\right),...,x_{5}^{32}\left(k\right)\right]\;\left(k=1,2,3,\ldots ,\mathrm{LN}∕32\right).$$

#### 2.4.2 Feature Compression

WPT normally generates a large number of features for classification. To reduce the workload of following classifier and prompt the proposed system to be real-time processing, the SVD technique is used in our system to extract the new feature from the *X* for reducing dimension. As a matrix factorization technique, SVD is proven to be reliable and efficient (38, 39). The feature matrix, *X_L_*_×_*_M_* (*L* = *LN*/32, *M* = 32), given by equation (10) is decomposed by the SVD technique given by the following equation:

(11) $$X_{L\times M}=U_{L\times L}\Lambda_{L\times M}V_{M\times M}^{T},$$

where *U* and *V* are the *L* × *L* and *M* × *M* orthogonal matrixes, and Λ is a *L* × *M* nonnegative diagonal matrix described as follows:

(12) $$\Lambda =\left(\begin{array}{c} S\;0\\ 0\;0 \end{array}\right),\;S=\mathrm{diag}\left(\sigma_{1},\sigma_{2},...,\sigma_{r}\right),$$

where *r* is the rank of the matrix, and *σ*_1_, *σ*_2_, … , *σ*_r_ are the singular values of matrix *X*, which are used as the extracted features. Since (*LN*/32) is normally larger than 32 in this article, the extracted features are with the size of 32.

#### 2.4.3 Feature Selection

There are 32 features from each MMG channel after the feature compression stage. However, these features have different degrees of relevance for the hand-motion classification. The MMG signal obtained by the MMG acquisition system contains both the mechanical signal generated by the hand motions and noises from different sources such as electrical noise and the vibration noise from the surroundings. The features containing the noise usually have an adverse effect for the recognition. It should be noted that a larger number of SVD features do not always lead to more effective classification results (24). Therefore, a feature ranking algorithm is required to select the most relevant features to further improve the recognition results.

In the proposed system, the methodology proposed in Ref. (40) is utilized for features ranking. The parameter *F*-value is calculated from the features extracted by WPT–SVD algorithm. For each feature, the bigger value of the *F* indicates higher importance for classification. The details of how to calculate the *F* are described following.

First of all, the average distance of each kind of feature from the same finger gesture is calculated by the following equation:

(13) $$d_{i,j}=\frac{1}{N\times (N-1)}\sum_{m,n=1}^{N}\;|p_{i,j}(m)-p_{i,j}(n)|;\;(m,\;n=1,\;2,\;\ldots ,\;N,\;m\neq n),$$

where *N* is the number of the samples of the same finger gestures, *p_i,j_*(*m*) is the *m*th sample of the *i*th feature of the *j*th gesture. For each feature, its average distance between 5 finger gestures can be defined as follows:

(14) $$d_{\mathrm{ai}}=\frac{1}{M}\sum_{j=1}^{M}d_{i,j}.$$

The *ai* is the number of the gestures. The value of *M* is 5 since there are five gestures needed to be recognized. Then, we calculate the average value of each feature in the same gesture as follows:

(15) $$p_{\mathrm{ai},j}=\frac{1}{N}\sum_{n=1}^{N}p_{i,j}(n)\;(\;n=1,\;2,\;\ldots ,\;N).$$

The average distance of each feature between different finger gestures can be defined by the following equation:

(16) $${d^{\prime }}_{\mathrm{ai},j}=\frac{1}{M\times (M-1)}\sum_{m,n=1}^{M}|p_{\mathrm{ai},m}-p_{\mathrm{ai},n}|\;(m,\;n=1,\;2,\;\ldots ,\;N;m\neq n).$$

Finally, the F-values can be calculated as follows: $F=d_{\mathrm{ai}}∕{d\prime }_{\mathrm{ai}}$. Following this algorithm, the 32 features are ranked in descending order according to its F-value.

### 2.5 Classification Module

The last module of our FGR system is the classifier to recognize the finger movement based on the features selected. In many reported MMG-based hand-gesture recognition systems, various pattern recognition algorithms were used such as linear classifier (24), multilayer perception (26), and neural networks (21). In our system, the classifiers, NBC, KNN, and SVM are selected, and the performance of three algorithms is compared.

#### 2.5.1 NBC

NBC, as a subclass of Bayes classification algorithm, is widely applied in the pattern recognition because of its simplicity and effectiveness. In some applications, its performance is comparable with other classifiers with increased computational complexity (41). Naive Bayes classification model assumes that each feature is independent for the classification. Although this unrealistic assumption limits its scope of applications, the time and space complexities are reduced. Therefore, the NBC is still a popular choice in various applications (42, 43). The implementation of NBC is based on the reference (44, 45).

#### 2.5.2 KNN

KNN is a classification model based on statistical analysis. The KNN algorithm classifies the sample according to the class of its k-nearest neighbor samples in the feature space. And the sample is assigned to the class where most of its neighbor samples belong to. KNN is a simple classifier without priori statistical knowledge. Thus, it is widely used in applications with non-normal or unknown sample distributions (46). However, its performance will be degraded when the number of samples is large (47). The implementation of KNN is based on the reference (48).

#### 2.5.3 SVM

SVM is a machine learning algorithm based on the statistical learning theory. It is good to deal with the situations when small samples are available and high dimension pattern recognition task is given (49). The libSVM3.12 package (50) is adopted for programming implementation in our system. The polynomial kernel function, *G*(*x*), given by equation (17) is utilized in SVM classifier,

(17) $$G\left(x\right)={\left(\gamma \times x\times z+\mathrm{coef}\;0\right)}^{\mathrm{degree}},$$

where *x* is the vector of input features, *z* is the support vector produced by SVM after training, and the parameters *coef*  0, *γ*, and *degree* are set to 0, 1, and 3, respectively, in our system.

## 3 System Characterization Results

In this section, we will describe the detailed experimental protocol for our FGR system characterizations. The characterization results are presented in this section as well with discussions. First of all, the performance of two TED algorithms introduced is compared with the manual event detection method. After that, the effectiveness of the features ranking and the performances of different classifiers are presented and discussed. At the end of this section, possible factors affecting the recognition results such as power of grip (PG) and body mass index (BMI) are discussed.

### 3.1 Participants

To fully characterize our system, twelve healthy participants (8 males and 4 females, 1 left-hander and 11 right-handers, age: 23 ± 3.21, height: 164.2 ± 7.65 cm, weight 66.2 ± 16.51 kg) participate in our experiments voluntarily. No medical history of neuromuscular disorders is reported. All the participants understand and agree with the experiment protocol before joining in the test for MMG signal acquisition.

### 3.2 Experimental Procedure

All participants are instructed to use five fingers of their dominant hand to tap, including thumb tapping, index finger tapping, middle finger tapping, ring finger tapping, and little finger tapping. Following a metronome with 30 beats per minute, every participant is asked to tap five fingers one by one from thumb to little finger, and each finger gesture is repeated five times before moving to next finger. The above process is repeated five times with 5 min interval to avoid the unreliable samples due to large number of consecutive tappings on the same finger. Therefore, there are totally 125 finger-gesture movements (5 finger gestures × 5 times × 5 rounds) for every participant. A total of 25 movements on each finger are obtained from tapping with either short time interval or long time interval which increases the variety of data set.

### 3.3 Characterization of Auto-Event Annotation Module

The auto-event annotation module in our system is tested. In details, the performance of two automatic TED algorithms is compared with the manual approach which is supposed to be optimal. The false detection events ratio (FDER) of the RMS-based and the DTF-based automatic TED algorithms are calculated which relates to the error rate of the muscle activity event detection. In addition, the accuracies of the MMG signal segmentation by different methods including DTF, RMS, and manual cutting are calculated and discussed.

#### 3.3.1 The Error Rate of Automatic TED

**Figure 3:**
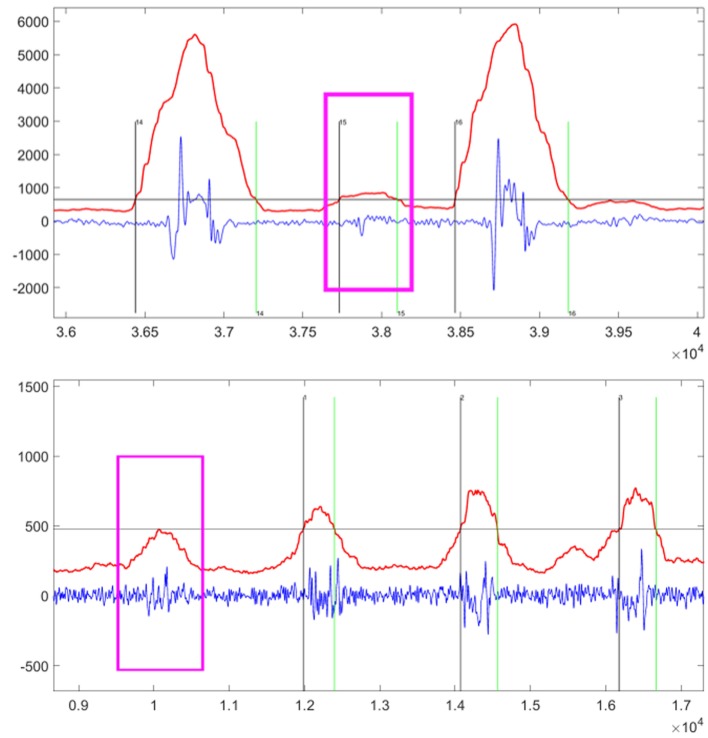
The examples of false negative event (top) and false positive event (bottom) highlighted by the pink windows. The vertical axis title is “Amplitude” and the horizontal axis title is “Number of Sampling Points.” The sampling rate is 1 kHz, and then the above chart shows segments lasting approximately 4 s (top) and 8 s (bottom). Within this chart, the blue line is the original MMG signal, the red line is the automatic TED result, and the horizontal gray line is the threshold calculated by equation (3) or equation (6). Thus, the vertical black line indicates the start point of detected event after which the red line goes beyond the gray line, while the vertical green line indicates the end point of detected event after which the red line goes under the gray line.

In this experiment, the false detection events in two scenarios are measured. The first one is the false negative event shown on the top of Figure 3. The muscular contraction event occurs but the TED algorithm cannot detect it. In this situation, the proposed system is not able to recognize finger-gesture movements. The other one is the false positive event shown in the bottom of Figure 3. The muscular contraction events can be detected by the TED algorithm while this signal segment contains irrelevant information. As a result, the wrong recognition results will be obtained by the proposed system.

FDER is a suitable indicator to measure and compare the performance between the RMS- and DTF-based automatic TED algorithms. The FDER is defined by the equation (18):

(18) $$\mathrm{FDER}=\frac{N_{\mathrm{FP}}+N_{\mathrm{FN}}}{N_{\mathrm{all}}},$$

where the *N_all_* is the number of all events detected by the algorithm. The *N_FP_* and *N_FN_* are the numbers of the false positive event and the false negative event, respectively.

In our experiment, all the data are processed separately by the RMS- and DTF-based automatic TED algorithms. The FDER values are 1.5 and 2.2%, respectively, for RMS-based method and the DTF-based method.

#### 3.3.2 The Accuracy of MMG Segmentation

**Figure 4:**
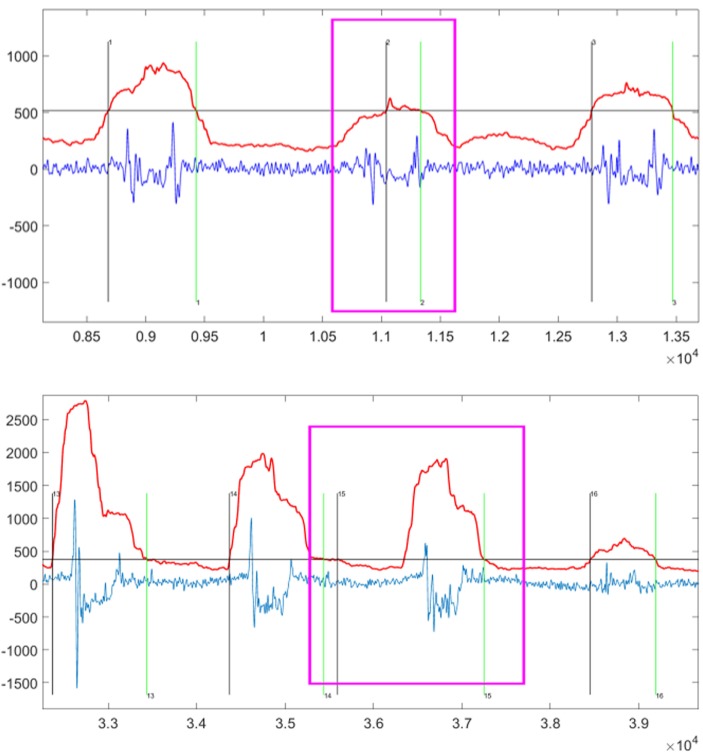
The examples of inaccurate extraction by automatic TED Algorithms highlighted by the pink windows. The vertical axis title is “Amplitude” and the horizontal axis title is “Number of Sampling Points.” The sampling rate is 1 kHz, and then the above chart shows segments lasting approximately 5 s (top) and 6 s (bottom). Within this chart, the blue line is the original MMG signal, the red line is the automatic TED result, and the horizontal gray line is the threshold calculated by equation (3) or equation (6). Thus, the vertical black line indicates the start point of detected event after which the red line goes beyond the gray line, while the vertical green line indicates the end point of detected event after which the red line goes under the gray line.

There are two tasks for the TED algorithms. The first task is to detect the fingers activity events correctly. The second task is to extract the MMG signal segment accurately. The FDER is the index to measure the performance of the first task. Next, we discuss how to measure the performance of the second task. There are two scenarios when the MMG signal segment is extracted inaccurately. As shown in Figure 4, the segment of the MMG signal shown in the top is very weak where the starting point of the event is too close to the end point, resulting in loss of the key information. On the other hand, the sub-figure in the bottom part of Figure 4 shows that the MMG signals contain some noises which may introduce some interferences. These inaccurate segmentations are the two factors that can degrade the recognition accuracy of the proposed FGR system.

**Figure 5:**
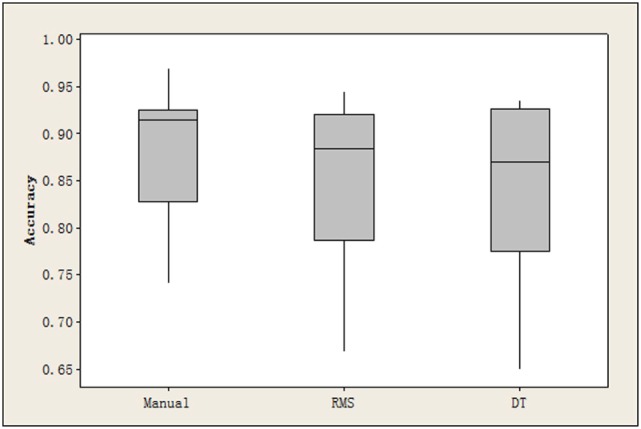
The recognition accuracy achieved by the manual segmentation, RMS- and DTF-based automatic TED algorithms, respectively.

Currently, there is no quantitative index to directly measure the quality of the MMG signal segment extraction. As our system focuses on the final finger-gesture recognition accuracy, we only compare the performance of RMS- and DTF-based TED algorithms with manual segmentation in achieving the final recognition rate. Among that, manual segmentation is supposed to be the accurate and optimal method for extracting the finger tapping event. In this comparison experiment, only the first 4 features after feature ranking process are used, and the NBC classifier is used. The results are reported in the Figure 5. This figure clearly shows that the manual segmentation has the best performance for the proposed FGR system, followed by the RMS- and DTF-based automatic TED algorithms.

#### 3.3.3 Discussion of TED Algorithm Evaluation

From the results in Section 3.3.1 and 3.3.2, the manual segmentation has slightly better performance compared with two automatic TED algorithms. If the performance between two automatic TED algorithms is compared, the RMS-based approach has a slightly better performance. However, the DTF-based approach has less computational complexity compared with the RMS-based method. Thus, the DTF-based approach is preferred in some low cost and real-time processing applications.

### 3.4 Classification Evaluation

The recognition accuracies of the classifiers SVM, KNN, and NBC are computed, and the results are compared and discussed. There are in total of 64 features from two-channel recorded MMG signals and 32 features are extracted from each channel. During the system characterization evaluation, the appropriate number of features used for recognition is also studied. Different numbers of features are selected by the ranking algorithm and the resulting recognition accuracies are compared. In this section, the manual segmentation for TED is selected to reduce unnecessary interference.

**Figure 6:**
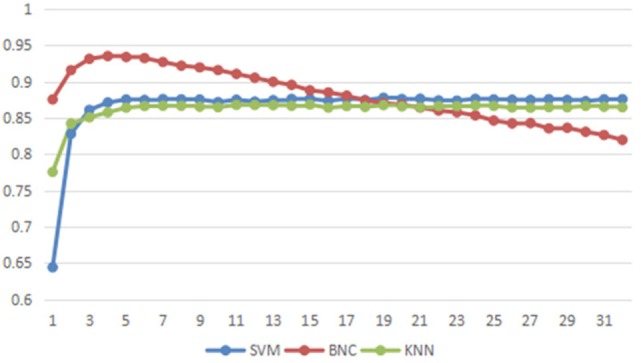
The accuracy of different classifier with different number of features. The X-axis is number of the features from each channel for classification. The Y-axis is the average accuracy of all participants.

The experimental results are shown in Figure 6. The accuracy rate of identification is up to 94.0%, and the average accuracy rate is 87.9% by the NBC. The highest recognition results (94%) in NBC classifier are obtained when the first 4 features of each channel are used. The accuracy is reduced with the increased number of features as NBC assumes that every feature is dependent. In other words, each feature has the same importance impact for the NBC classifier. According to the ranking algorithm introduced in Section 2.4.3, the feature with lower rank means that it is less useful because of more noises involved. Thus, when less irrelevant features are used in the classification process, more noises are introduced, resulting in less accuracy of the NBC classifier. In addition, this result also shows that the feature ranking algorithm is critical for the NBC-based classifier, because it can remove the irrelevant features and improve the NBC performance.

The recognition accuracies from the KNN and SVM classifiers are improved slightly when the number of features per channel is larger than 3. The possible reasons of this observation are explained as follows. KNN is a classification algorithm based on the distance of the feature space. In that case, it is insensitive to the weak features. Therefore, the classification accuracy is not degraded when the number of weak features is increased (46). The SVM classifier aims to optimize the performance based on all features, which definitely includes the weak features. The best linear combination of all features is chosen by the SVM classification. Thus, the SVM performance is becoming slightly better when more features are used in Figure 6. In addition, the performance of SVM is also dependent on the used kernel function. The proposed system only considers the polynomial kernel and nonlinear kernel functions may improve the performance which will be explored in the future work.

### 3.5 Influencing Factors

As the MMG signals are produced by the muscle and transmitted by the soft tissue (14), intuitively, our FGR system might have better performance on participants who have well developed forearm muscles and thinner hypodermic fat. To verify this hypothesis, the power of grip (PG) is measured as the index of muscle strength, and the body mass index (BMI) is recorded as the index of hypodermic fat for each participant in this experiment. The recognition performance on each participant is then compared.

**Figure 7:**
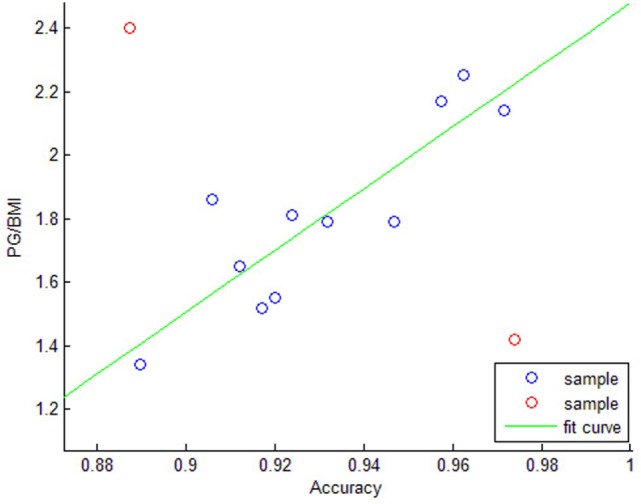
The relationship between the recognition accuracy and PG/BMI value. The two red samples are outliers, while the blue samples have a pronounced linear relationship.

The Figure 7 shows the relationship between (*PG*/*BMI*) and the recognition accuracy rate. The X-axis is the recognition accuracy based on every participant data where the NBC classifier with 8 features (4 features for each channel) is adopted for the FGR system. The Y-axis is the value of PG divided by BMI, namely (*PG*/*BMI*). There is a linear relationship between the (*PG*/*BMI*) and the recognition accuracy in our experiments, and the cross-correlation coefficient is 0.728. The above results show that the recognition accuracy rate of FGR system is proportional to the muscle strength, while has a inverse proportion to the hypodermic fat. Although more testing data are needed to reach a firm conclusion instead of the above results from 12 participants, it is highly possible that the recognition accuracy of FGR system and the value of (*PG*/*BMI*) have a linear relationship. The MMG-based FGR system may have higher recognition accuracy on participants with higher ratio of PG and BMI. This finding also suggests that the proposed FGR system users should take more exercises to strengthen the forearm muscles and reduce the hypodermic fat to well control the system. This system may be adopted in different applications such as prosthetic control.

## 4 Conclusion

In this article, a novel motion-intend-based finger gesture recognition system based on mechanomyogram (MMG) signal is presented to recognize each finger tapping. This is the first system capable of detecting every finger tapping. The architect and the design of the system are presented. A full system characterization is also evaluated and the recognition accuracies of the system are compared under different experimental settings. Our system is able to achieve up to 94% accuracy. How different factors such as PG and BMI of the participants affect the system performances are also discussed, and the obtained results suggest that the proposed FGR system users should take more exercises to strengthen their muscles and reduce the hypodermic fat.

The proposed system focuses on identifying motion intentions instead of actual finger movements. It is therefore suitable for finger rehabilitation training of stroke patients. In some cases, when stroke patients try to move their fingers, they cannot find corresponding action. With the proposed system, we can provide guidance to the patients by correcting the effort made toward their intentions of finger movement and can further quantitively measure the accuracy by exploring the enhancement of the system design. The quantified result is beneficial to further understanding of training intensity, contrasting training effect, and so on. Therefore, applying the proposed system to the stroke rehabilitation is worthy of continuous exploration and development.

## Ethics Statement

This study was carried out in accordance with the recommendations of Ethical Committee of Health Science Center, Shenzhen University with written informed consent from all subjects. All subjects gave written informed consent in accordance with the Declaration of Helsinki. The protocol was approved by the School of Biomedical Engineering, Health Science Center, Shenzhen University, China.

## Author Contributions

All the authors listed in this article have participated in the following work: substantial contributions to the conception or design of the work; or the acquisition, analysis, or interpretation of data for the work; drafting the work or revising it critically for important intellectual content; final approval of the version to be published; and agreement to be accountable for all aspects of the work in ensuring that questions related to the accuracy or integrity of any part of the work are appropriately investigated and resolved. HD and QH designed and implemented the simulation model, and prepared the manuscript; YZ built initial constructs and GD supervised the project; SC supervised the analysis and edited the manuscript; all the authors discussed the results and implications and commented on the manuscript at all stages.

## Conflict of Interest Statement

The authors declare that the research was conducted in the absence of any commercial or financial relationships that could be construed as a potential conflict of interest.
